# The effect of education given before surgery on self-esteem and body image in women undergoing hysterectomy

**DOI:** 10.4274/tjod.95770

**Published:** 2015-12-15

**Authors:** Şengül Yaman, Sultan Ayaz

**Affiliations:** 1 Gazi University Faculty of Health Sciences, Department of Nursing, Ankara, Turkey

**Keywords:** Body image, self-esteem, health education, hysterectomy

## Abstract

**Objective:**

To evaluate the effect of information provided before surgery on the self-esteem and body image of women undergoing hysterectomy.

**Materials and Methods:**

The study had a semi-experimental design with pre-post tests. A total of 60 women were included in the study and divided into two groups, the intervention group (n=30) and control group (n=30). A questionnaire, the Rosenberg self-esteem scale, and the body image scale were used to collect data.

**Results:**

The pre- and post-test body image scores were similar in the intervention group patients, but the post-test scores were significantly higher in the control group (p<0.05). The pre- and post-test self-esteem scores were again similar in the intervention group, but the post-test scores were significantly lower in the control group (p<0.05).

**Conclusion:**

This study revealed that health education given to patients prior to hysterectomy protects body image and consequently self-esteem.

## INTRODUCTION

Female genital organs are very important in determining female sexual identity. For many women, the uterus is the symbol of femininity, sexuality, fertility, and motherhood, and they feel that their partners will not like them, their attractiveness will decrease, and they will be unable to fulfill sexual function after surgery. Women are also afraid that their sexual desire will decrease and early aging will follow after removal of their ovaries^(1,2)^.

Hysterectomy surgery is commonly performed in gynecology. Hysterectomy causes physical and biologic effects such as disruption of body integrity and loss of fertility, and may also cause psychosexual and social problems such as loss of the love of others and feminine features. Most women perceive the uterus as an incubator to carry babies and believe that it is a symbol of their fertility. For example, many women believe that the best period of their lives will come to an end following hysterectomy and view the surgery as the loss of youth, femininity, and health^(3)^.

Women who were provided information regarding conditions they may experience after hysterectomy and discharge were reported to feel better and experience higher quality of life than women who did not receive such information^(4)^. The importance of health education services preoperatively, at discharge, and postoperatively has been emphasized and this service should be provided by expert nurses^(5)^.

This study was performed to compare pre- and post-test values in mean body image and self-esteem test scores of patients were provided information with those of controls. The aim of present study was to evaluate the effect of the education given before surgery on self-esteem and body image in women undergoing hysterectomy.

## MATERIALS AND METHODS

### Design and sample

This study had a semi-experimental design with pre-post tests. The study population consisted of women undergoing hysterectomy as inpatients at Gazi University Hospital’s, Gynecology Clinic. Power analysis was conducted using PASS 2008 software to determine sample size to achieve 80% power with an α of 0.05, based on a meaningful difference of 0.07 between the study groups. The calculation showed that a minimum of 24 subjects was required. A total of 60 women were included in the study. The women were divided into two groups, the intervention (n=30) and control group (n=30) subjects each in the groups. Women were included in the study if they could communicate, were undergoing hysterectomy for benign disease, were not menopausal, and had no history of oophorectomy.

### Data collection

A questionnaire, the Rosenberg self-esteem scale, and the Body image scale (BIS) were used to collect data.

Closed-ended questions were used in the questionnaire to determine sociodemographic characteristics such as age, education level, marital status, employment status, having children, and wanting more children.

Secord and Jourard developed the BIS in 1953. The scale aims to evaluate how much the subject is pleased with the various parts and functions of the body. The Turkish validity study for the scale was conducted by Hovardaoglu^(6)^ in 1993 and the Cronbach alpha internal consistency coefficient was found to be 0.91 (p<0.01). The scale consists of 40 items. Each item is about an organ or body part (e.g. arms, legs, face,) or a function (such as the level of sexual activity). Each item is scored from 1 to 5 and example response options are “I like it very much” (1 point), “I like it a lot” (2 points), “I cannot decide” (3 points), “I do not like it” (4 points) and “I do not like at all” (5 points). The total score of the scale ranges from 40 to 200 and there is no cut-off point. High scores indicate an increase in dissatisfaction.

The Rosenberg self-esteem scale was developed by Rosenberg in 1965. The Turkish validity and reliability study was conducted by Cuhadaroglu^(7)^ and the validity coefficient was found to be 0.71. The reliability coefficient was 0.75 on the basis of the test-retest reliability method. The scale consists of a total of 63 multiple-choice questions in 12 sub-categories. The first 10 items of the scale were used to measure self-esteem for the purposes of this study. Each item has response options such as “Very true”, “True”, “Wrong” and “Very wrong”. A total score from the first 10 questions of 0-1 indicates high, 2-4 moderate, and 5-6 low self-esteem.

### Procedure

In the intervention group, the questionnaire and scales were administered before surgery to all subjects who participated in the study. The women in the intervention group received the standard of nursing care provided at the service, as well as a training guide prepared before surgery, and health care information. The educative contents were the structure and functions of the female reproductive organs, the reproductive cycle, menopause, the type of surgery and its postoperative effects on sexuality. Scales were again administered to the women one week after surgery. The post-tests were conducted one week after the training in order to determine the effect of training and changes in the mean scores of patients regarding body image and self-esteem. The reasons for this time selection were that postoperative problems were at least seen or not seen, and also that the information that had been given was not forgotten.

In the control group, the questionnaire and scales were administered to the women in one week before and after surgery. This group only received the standard of nursing care provided at the service.

### Ethical aspects of the study

Written permission was obtained from the head physician of the hospital before the study. The informed consent form was read to the participating women and their written and verbal consent was obtained. The relevant information was provided to the patients in the control group after the final tests were administered.

### Data evaluation

Statistical analyses were performed using SPSS version 13.0 software (SPSS Inc, Chicago, IL). Categorical variables were presented as frequencies and percentages. For the comparisons between groups, the Chi-square test was used for categorical variables. Continuous variables are expressed as mean ± standard deviation. Continuous variables were compared using the independent Sample t-test or paired Sample t-test. Independent Sample t-test was used to compare the difference between pre- and post-test scores of patients in the intervention and control groups. Paired Sample t-test was used to compare the difference between pre- and post-test scores of patients in each group. A two-sided p value <.05 was considered significant for all analyses.

## RESULTS

Table 1 shows the comparison of descriptive characteristics of the patients in the intervention and control groups. Descriptive characteristics were not statistically significantly different between the groups (p>0.05). Of the patients in the intervention group, 50.0% were elementary school graduates, 80.0% were married, and 83.3% had children. Of the patients in the control group, 76.7% were elementary school graduates, 90.0% were married, and 93.3% had children (Table 1).

The mean and standard deviations of body image scores are shown in Table 2. A comparison of the patients’ mean pre- and post-test scores in the intervention and control groups revealed that the pre- and post-test BIS were similar in the intervention group, but the post-test scores were significantly higher in the control group (p<0.05) (Table 2).

The mean and standard deviations of self-esteem scores are shown in Table 3. A comparison of the mean pre- and post-test scores of patients in the intervention and control groups revealed that the pre- and post-test self-esteem scores were similar in the intervention group (p>0.05), but the post-test scores were significantly decreased in the control group (p<0.05) (Table 3).

## DISCUSSION

Body image reflects a psychologic experience and is focused on a person’s feelings and thoughts. Although body image has a physiologic basis, it relates to physical, psychologic and, social experiences^(8)^. Alterations in body image can occur naturally, inadvertently or accidentally. A conflict in a woman’s image of her body and the image of her body in her mind creates a threat to her current body image^(9,10)^. Disease or absence of reproductive organs may translate into a feeling of deficiency as a woman^(11)^. The pre- and post-test body image scores of the patients in the intervention group were similar, but the post-test scores were higher in the control group. Our findings demonstrate that the body image of women in the control group decreased due to the removal of the uterus. The loss of the uterus can be perceived as a loss of femininity and vitality^(12)^. However, no change was observed in the body image of women in the intervention group. This is probably the result of explaining the effect of removing the uterus on sexuality and menopause with the educational content. Studies report that abdominal hysterectomy does not have an important effect on sexual function when the vagina is not excessively shortened and the ovaries are protected^(13,14,15)^.

Self-esteem is a concept that shows how much an individual loves, accepts, and respects themself. This is a learned image that is influenced by the social environment (mainly family)^(16)^. A complicated relationship is present between self-esteem and body image. Low self-esteem stems from a negative body image in some women, and can lead to a negative body image in others^(17)^. There were similar pre- and post-test self-esteem scores among the patients in the intervention group but lower post-test self-esteem scores in the control group. This finding can be explained by the effect of the pre-operative training provided in the intervention group. It can be stated that the intervention groups’ body images did not change and therefore their self-esteem was not negatively affected because of the training. The deteriorated body image after surgery in patients in the control group is thought to have affected their self-esteem. A reduction in self-esteem is possible in women (social environment effect) who are afraid that they would not be liked due to a deteriorated body image.

## CONCLUSION

This study revealed that health education given to patients prior to hysterectomy protects body image and consequently self-esteem. Therefore, the scope of nursing care provided in gynecology clinics should definitely include providing health education towards protecting body image and self-esteem.

## Figures and Tables

**Table 1:**
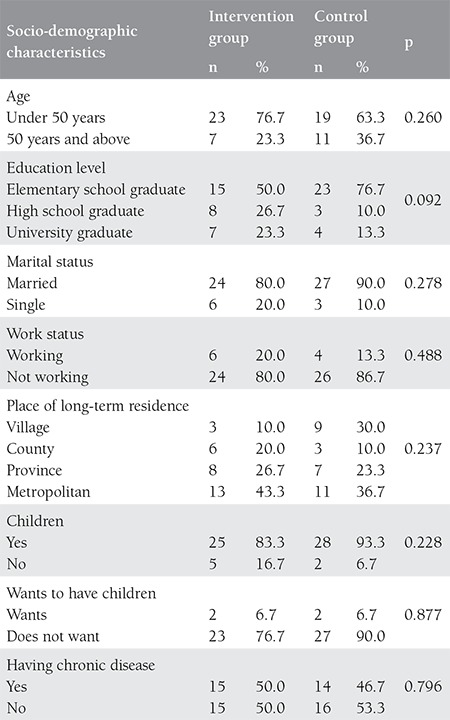
Socio-demographic characteristics of patients

**Table 2:**
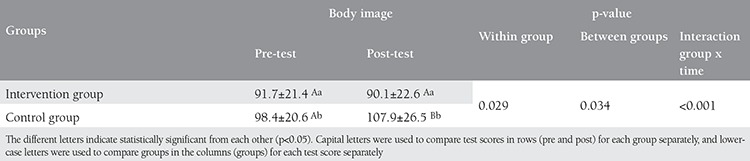
Mean body image scores of patients before and after education

**Table 3:**
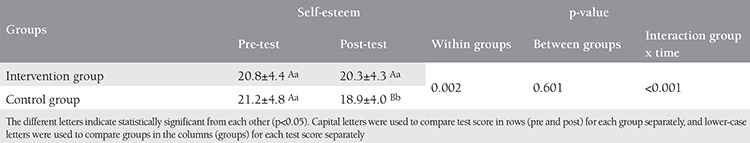
Mean self-esteem scores of patients before and after education
